# Fertility Awareness Methods: Distinctive Modern Contraceptives

**DOI:** 10.9745/GHSP-D-15-00297

**Published:** 2016-03-25

**Authors:** Shawn Malarcher, Jeff Spieler, Madeleine Short Fabic, Sandra Jordan, Ellen H Starbird, Clifton Kenon

**Affiliations:** a United States Agency for International Development, Office of Population and Reproductive Health, Washington, DC, USA; b Independent Consultant, Washington, DC, USA

## Abstract

Fertility awareness methods—the Lactational Amenorrhea Method, the Standard Days Method, and the Two Day Method—are safe and effective, and they have important additional benefits that appeal to women and men. Including these modern contraceptives in the method mix expands contraceptive choice and helps women and men meet their reproductive intentions.

This peer-reviewed commentary represents the technical position of the Office of Population and Reproductive Health of the United States Agency for International Development.

Fertility awareness methods (FAMs), comprising the Lactational Amenorrhea Method (LAM), the Standard Days Method (SDM), and the Two Day Method (TDM), are often left out of the basket of contraceptive options for women and couples because governments, donors, policy makers, and providers perceive them as inferior contraceptive methods. When FAMs are offered as an option, FAM users may be incorrectly lumped with traditional method users in reports and data analyses. Notably, a recent unpublished review of contraceptive method classification commissioned by the World Health Organization (WHO) found that LAM was deemed a “traditional” rather than a “modern” method in 2 of 5 major family planning publications or data-reporting sources. SDM was included as a modern method in only 3 of 6 publications, and the Two Day Method appeared in only 1 publication.

## WHY DO WE CARE?

Fertility awareness methods are commonly misperceived as traditional methods and thus are often left out of family planning programming.

*“What gets measured gets done*.” As governments and donors recommit themselves to advancing the rights of women and girls to decide—freely and for themselves—whether, when, and how many children to have, more attention is cast on the key measures of success laid out at the London Summit on Family Planning in 2012. As agreed on by committed governments and donors, key indicators of success explicitly identify *modern* method use as the outcome of interest. In this context, common misperceptions that FAMs are traditional methods mean that countries do not prioritize investments in their introduction or expanded provision. These methods may be absent from training curricula, counseling materials, logistics systems, and procurement processes. FAM users may be left out of key monitoring and data collection activities. If users of FAMs are not counted as modern contraceptive users, we as a community are failing to recognize a valid and important choice to meet client needs—*what gets counted gets supported*.

## WHAT MAKES FAMS “MODERN”?

The United States Agency for International Development (USAID) supports FAMs as modern contraceptives because these methods meet the criteria for a modern contraceptive. FAMs:

- Are effective at pregnancy prevention,
- Are safe,
- Are based on a sound understanding of reproductive biology,
- Include a defined protocol for correct use, and
- Have been tested in appropriately designed studies to assess effectiveness under various conditions.

The Standard Days Method is an easy way to track the fertility cycle and fertile window for women whose menstrual cycle lengths are 26–32 days. The SDM algorithm is based on data from 7,500 cycles collected as part of a 5-country clinical trial supported by WHO. A second clinical trial of SDM followed, which documented a 95% effectiveness rate in perfect use and an 88% typical use rate; the typical use rate is commensurate with barrier methods, including male and female condoms and diaphragms.^1^^,^^2^

The Two Day Method is a modification of the Billing's Ovulation Method and relies on a woman’s assessment of her cervical secretions to identify her fertile days. The algorithm is based on data from multiple, large data sets. Clinical trials following more than 400 women for 13 cycles of method use showed an efficacy rate of 96% with perfect use and over 86% with typical use, also commensurate with barrier methods.^3^

The Lactational Amenorrhea Method, based on the fertility-suppressing effects of breastfeeding, requires that a woman be within 6 months postpartum, exclusively or nearly exclusively breastfeeding, and amenorrheic.^4^ Multiple studies have documented an efficacy of 99% in perfect use and 98% at 6 months in typical use, commensurate with effectiveness of injectables and combined oral contraception.^5^

## ADDITIONAL BENEFITS OF FAMS

Fertility awareness methods are knowledge-based, relying on women’s understanding of their fertile cycle and, in the case of LAM, of how breastfeeding practices can temporarily suppress fertility. In addition to offering a safe and effective alternative to other contraceptive methods, FAMs offer several additional benefits:

- They do not require clinical intervention, such as hormones, devices, or procedures.
- They are controlled by a woman and her partner.
- They increase a woman’s understanding of her fertility and biological processes.
- In the case of SDM and TDM, they provide the opportunity to facilitate pregnancy planning.
- FAMs can be offered through a wide variety of channels, including settings completely outside the health system.

Fertility awareness methods do not require clinical intervention and can be offered through a variety of service delivery channels.

Technical experts report that providers trained in FAMs appreciate their improved capacity to explain the basic concepts of fertility to their clients. This knowledge helps reassure their clients about the safety and efficacy of contraceptive methods generally, which may lead to increased acceptance of family planning. Providers who counsel on methods that require awareness and involvement of both partners, such as SDM, also develop the capacity to discuss relationship dynamics and partner communication—valuable skills regardless of the contraceptive method chosen by clients.^6^

USAID’s family planning programs are guided by the principles of voluntarism and informed choice. That is, every individual has the right to choose the number, timing, and spacing of her/his children; to choose freely whether and when to use contraception; and to choose from a broad range of contraceptive methods, with ample information about use, advantages, and side effects. Evidence of USAID’s commitment to these principles is its support for the development, testing, refinement, and introduction of new and improved contraceptive methods as well as its support for family planning program components that make that choice real. USAID aims to help countries meet the contraceptive needs of their people. Key to achieving this aim is expanding access to a wide and diverse method mix. USAID continues to support countries and the global family planning community in their efforts to incorporate modern methods, including FAMs, into the contraceptive method mix. The more contraceptive choice we can afford to women and men, the better equipped they will be to achieve their reproductive intentions. Key to expanding contraceptive choice is overcoming misconceptions. Let’s recognize FAMs for what they are: effective, safe, modern contraception.

USAID supports fertility awareness methods as modern contraceptives.

## Notes

**Peer Reviewed**
